# Global Climate Niche Estimates for Bioenergy Crops and Invasive Species of Agronomic Origin: Potential Problems and Opportunities

**DOI:** 10.1371/journal.pone.0017222

**Published:** 2011-03-09

**Authors:** Jacob N. Barney, Joseph M. DiTomaso

**Affiliations:** 1 Department of Plant Pathology, Physiology, and Weed Science, Virginia Polytechnic Institute and State University, Blacksburg, Virginia, United States of America; 2 Department of Plant Sciences, University of California Davis, Davis, California, United States of America; University College London, United Kingdom

## Abstract

The global push towards a more biomass-based energy sector is ramping up efforts to adopt regionally appropriate high-yielding crops. As potential bioenergy crops are being moved around the world an assessment of the climatic suitability would be a prudent first step in identifying suitable areas of productivity and risk. Additionally, this assessment also provides a necessary step in evaluating the invasive potential of bioenergy crops, which present a possible negative externality to the bioeconomy. Therefore, we provide the first global climate niche assessment for the major graminaceous (9), herbaceous (3), and woody (4) bioenergy crops. Additionally, we contrast these with climate niche assessments for North American invasive species that were originally introduced for agronomic purposes as examples of well-intentioned introductions gone awry. With few exceptions (e.g., *Saccharum officinarum*, *Pennisetum purpureum*), the bioenergy crops exhibit broad climatic tolerance, which allows tremendous flexibility in choosing crops, especially in areas with high summer rainfall and long growing seasons (e.g., southeastern US, Amazon Basin, eastern Australia). Unsurprisingly, the invasive species of agronomic origin have very similar global climate niche profiles as the proposed bioenergy crops, also demonstrating broad climatic tolerance. The ecoregional evaluation of bioenergy crops and known invasive species demonstrates tremendous overlap at both high (EI≥30) and moderate (EI≥20) climate suitability. The southern and western US ecoregions support the greatest number of invasive species of agronomic origin, especially the Southeastern USA Plains, Mixed Woods Plains, and Mediterranean California. Many regions of the world have a suitable climate for several bioenergy crops allowing selection of agro-ecoregionally appropriate crops. This model knowingly ignores the complex biotic interactions and edaphic conditions, but provides a robust assessment of the climate niche, which is valuable for agronomists, crop developers, and regulators seeking to choose agro-ecoregionally appropriate crops while minimizing the risk of invasive species.

## Introduction

The global energy sector is trending toward incorporation of increasing amounts of renewable energy, of which bioenergy—energy yielded from biological sources—is a growing component [1]. The United States (US) currently produces 4% (3.2 EJ) of its total energy from biomass [2], but has mandated 136 billion liters of renewable liquid transportation fuels by 2022, which may require up to 60 million additional hectares of land [3]. This additional cropland will not be evenly distributed across the US due to climatic variation, land availability, and resource requirements (e.g., irrigation). The US Department of Agriculture (USDA) estimates that nearly 50% of the biomass needed to meet the Renewable Fuel Standard will be grown in the Southeast, with an additional 43% in the Central-Eastern US [4]. However, identifying crops capable of producing high yields on marginal lands or degraded soils with minimal inputs will be a tremendous challenge to the sustainability of the bioenergy industry globally [2].

Identifying regions and the climatic suitability of proposed biofuel species within targeted regions will aid selection of the most appropriate bioenergy crops that require the fewest inputs. For example, the highest recorded yields occur in the Amazon floodplain for *Echinochloa polystachya* (100 MT ha^−1^ yr^−1^) and *Pennisetum purpureum* (88 MT ha^−1^ yr^−1^) [1]. Perennial grasses using the C_4_ photosynthetic pathway—*Panicum virgatum* (switchgrass), *Miscanthus* spp., *Saccharum* spp. (sugarcane), and *Pennisetum* spp.—are intrinsically nutrient, light, and water use efficient, especially in the humid warm regions of the globe. Additionally, fast growing trees that are harvested or coppiced on short rotations have the potential to provide high quality biomass [5]. Several studies have provided yield estimates or habitat suitability of select crops in certain parts of the world [e.g.], [ 1], [6], [7], [8,9], which begins to address the need for choosing appropriate crops that require minimal inputs. However, no global assessment of large-scale suitability for a variety of herbaceous, grass, and woody species has been conducted.

One additional complicating factor the bioenergy industry faces in achieving agronomic and economic goals is to prevent unintentionally introducing invasive species to susceptible natural or managed ecosystems [10]. The desirable traits that bioenergy crops must possess—rapid growth rates, high annual yields with minimal inputs of pesticides, fertilizers, and irrigation, tolerance of poor growing conditions—typify the invasive species ideotype [10], [11]. In fact, some of the taxa undergoing agronomic field trials are known invasive species in some portion of their introduced range [12], and exhibit an unknown risk to other environments [10]. Some attempts have been made to evaluate the risk posed by some bioenergy crops in their target region by using the Pheloung Weed Risk Assessment (WRA) model [10], [13], [14], [15], which includes, in part, an assessment of climatic suitability [16]. The authors have consistently found that the majority of the proposed bioenergy crops present an unacceptable level of invasion risk in their respective target regions according to this risk assessment. However, this WRA was designed as a pre-introduction evaluation for plants that are largely introduced for ornamental or horticultural purposes, and as such may be less relevant for bioenergy crops, or worse, may needlessly restrict adoption of “safe” crops due to misuse of an inappropriate risk assessment [10]. Additionally, one critical, yet almost always overlooked aspect of a risk assessment, is the evaluation of suitable habitat [10]. There is no possibility for invasion if the climate of the target region is unsuitable [17]. Similarly, there is no possibility for agronomic production if the climate is not suitable.

In the case of bioenergy crops, the climate niche represents both the region (possibly) suitable for agronomic production, as well as the regions (possibly) suitable for establishment outside of cultivation [18]. For example, the Southeastern US was the focus of kudzu (*Pueraria montana* var. *lobata* (Willd.) Maesen & S. M. Almeida ex Sanjappa & Predeep) introduction for soil stabilization and forage in the early 20^th^ century, as this region was climatically suitable based on the native range in Japan [19]. The favorable climate of the Southeastern US did not provide a barrier to surviving outside cultivation [20], while the originally desirable characteristics of rapid establishment and high growth rates contributed to the ultimate invasion of kudzu over 2.8 million hectares [19]. Therefore, comparing the climate niche of invasive species of agronomic origin with the climate niche of bioenergy crops may elucidate patterns and regions that could be the focus of screening and monitoring for escapes.

This study aims to provide global climate niche estimates for the leading bioenergy crops, as well as for invasive species that have an agronomic origin (i.e., were introduced as a forage or agricultural crop). Our objectives were to: 1) evaluate the global climate niche for grass, herbaceous, and woody bioenergy crops, 2) compare the bioenergy crop climate niche with invasive species that were widely introduced for agronomic purposes, and 3) compare the ecoregional distribution of both bioenergy crops and invasive species in North America, especially the continental US.

## Materials and Methods

### Species data

Estimating the fundamental niche, or climate-driven range, can be performed using the native range, introduced range, or both with various advantages and disadvantages [21], [22]. Using only the native range is the most restrictive estimate, but likely ignores the boundaries of the climate niche, which could only be elucidated by using the introduced range. Alternatively, the entire range (native+introduced) will best estimate a species fundamental niche at the risk of over-estimating the range potential in regions where the taxon is not yet introduced [21]. Since our goal was to provide a conservative estimate of the range potential for each species we chose to use the entire range (i.e., native and introduced) in our modeling.

The Global Biodiversity Information Facility (www.gbif.org) hosts a data portal of natural history collections across the globe, which is available for download. We accessed the portal (February 2010) for each species in our study, which primarily comprises herbarium collections with label data, and used only those collections with geolocations. Population location files ranged from 93 to 58,115 records with widely introduced crops (e.g., *Medicago sativa*) and weeds (e.g., *Schedonorous phoenix = Festuca arundinacea*) having the most collections, and more recently introduced species (e.g., *Jatropha curcas*) having many fewer recorded populations. Collections included wild, cultivated, ornamental, and irrigated locations, which were taken into consideration while fitting the model (ie, cultivated locations were not used to guide fitting the climate niche as cultivation can mitigate environmental stochasticity [20]).

### CLIMEX

We used the CLIMEX software to estimate the fundamental niche for each species, which utilizes the distribution and abundance of known populations to parameterize a climatic model [23]. CLIMEX is flexible in allowing model parameterization by visually matching the output to conform to the known distribution, while also allowing basic biological information to drive parameter estimation [see 18 as an example]. CLIMEX calculates a growth index where population growth is positive, and a stress index where population growth declines or is zero, each of which comprises sub-indices, based on the input parameters and climate [23]. The Ecoclimatic Index (EI) is the synthetic measure of the growth and stress indices and ranges between 0 and 100. Regions with an EI≤10 are very stressful and unlikely to support a population, while an EI>20 is favorable for population growth and an EI>30 represents a region able to support substantial population densities [24], [25], [26].

For this study, model output was visually estimated to match the current distribution (i.e., high EI values where population density is highest, and low EI values where no known populations exist). Parameters were subsequently refined using biological information, if any existed, from the primary literature. The parameters were then adjusted iteratively to yield a model that most closely matches the distribution and abundance of both native and introduced populations globally, while always attempting to minimize overestimation. Therefore, we set a threshold of ≥80% of GBIF collections must occur within ‘favorable’ to ‘very favorable’ regions (i.e., EI≥20). Many species are current crops (e.g., turfgrasses, agronomic crops, ornamental plantings) that receive irrigation in some portion of their range, or species that occur in riparian areas (e.g., *Arundo donax*, *Phalaris aquatica*). Therefore, in most cases a second simulation was run with the “Permanent Water Scenario” by adding 9.6 mm day^−1^ to simulate the effects of agronomic irrigation or areas with a perennial source of water (e.g., streams, irrigation canals, and wetlands) [18], [27]. Regions with an EI≥20 from the irrigation scenario were added to the final maps to exhibit regions suitable with a water subsidy. To create niche maps, CLIMEX results were exported to the geographic information system Manifold 8.0 (Carson City, NV) where Kriging was performed and contours generated for each EI level. The area of each contour was not calculated, as this value would be a gross overestimate of the actual range potential of each species because it represents the fundamental niche, which does not consider biotic interactions, edaphic conditions, disturbance regimes, land use, or trophic dynamics.

One of our ultimate objectives relates to using the niche maps for risk assessment at sub-national boundaries. We have chosen ecoregions, which represent regions of repeating patterns of characteristic associations of soil and landforms that include the biota (including humans), geology, physiography, hydrology, and climate, at the scale of interest [28]. The International Commission for Environmental Cooperation (www.cec.org) delineated three levels of ecoregions, of which we are using Level II, which comprises 50 types in North America and 20 in the Continental US, and best captures the desired level of spatial scale and utility. This ecoregional designation seems most appropriate given the scale, as well as being promoted by the US Environmental Protection Agency (EPA) (www.epa.gov/wed/pages/ecoregions/na_eco.htm). CLIMEX niche maps were overlaid on the Level II ecoregions in Manifold and the number of GBIF populations contained within each ecoregion were calculated, as was the presence of ‘very favorable’ habitat (EI≥30) for each species.

## Results

The CLIMEX model performed well, achieving ≥80% inclusion of global populations at an EI≥20 for all bioenergy crops and invasive species (Tables 1, 2, 3). Model accuracy was positively correlated with the number of global population records (P = 0.072), especially for the forage species *Dactylis glomerata*, *Elytrigia repens*, *Medicago sativa*, *Phalaris arundinacea*, and *Schedonorus phoenix*, which had records ranging from 24,978 to 58,115.

**Table 1:** CLIMEX parameters and values, number of records used in the analysis, and model accuracy for the eight perennial and one annual (*Sorghum bicolor*) grass biofuel feedstock crops.

Parameter	*Arundo donax*	*Miscanthus×giganteus*	*Miscanthus sacchariflorus*	*Miscanthus sinensis*	*Panicum virgatum*	*Pennisetum purpureum*	*Phalaris arundinacea*	*Saccharum officinarum*	*Sorghum bicolor*
DV0	10°C	8°C	10°C	10°C	10°C	15°C	5°C	15°C	5°C
DV1	20°C	16°C	15°C	20°C	20°C	25°C	8°C	23°C	12°C
DV2	35°C	30°C	28°C	30°C	30°C	40°C	27°C	33°C	34°C
DV3	40°C	35°C	30°C	35°C	35°C	42°C	30°C	36°C	40°C
SM0	0.1	0.19	0.1	0.1	0.1	0.2	0.1	0.35	0.01
SM1	0.2	0.3	0.2	0.2	0.2	0.7	0.4	7	0.1
SM2	2	1	1	1	1	1.5	2	1.5	0.6
SM3	10	10	10	10	10	2.5	10	10	10
TTCS	0°C	0°C	−5°C	0°C	10°C	10°C	-	6°C	−3°C
THCS	−0.0005	−0.0003	−0.0003	−0.0003	−0.00001	−0.0009	-	−0.01	−0.0005
TTHS	40°C	35°C	32°C	35°C	35°C	42°C	40°C	40°C	45°C
THHS	0.002	0.01	0.05	0.01	0.01	0.0002	0.002	0.0002	0.005
SMDS	0.01	0.1	0.1	0.1	0.1	0.1	0.02	0.25	0.01
HDS	−0.005	−0.02	−0.009	−0.02	−0.02	−0.0001	−0.005	−0.01	−0.0005
SMWS	-	-	-	-	-	2.5	-	-	-
HWS	-	-	-	-	-	0.002	-	-	-
TTHW	35°C	33°C	35°C	35°C	-	-	-	-	-
MTHW	1	0.5	0.5	0.5	-	-	-	-	-
PHW	0.075	0.05	0.05	0.05	-	-	-	-	-
*N*	6819	-	142	338	1714	593	49996	93	1096
EI≥30	98.7%	-	90.1%	90.2%	78.7%	85.0%	99.0%	77.4%	94.0%
EI≥20	99.3%	-	97.9%	97.3%	87.4%	91.2%	99.4%	79.6%	97.2%
EI≥20+water	-	-	97.9%	97.3%	96.6%	-	99.8%	87.1%	-

**Table 2:** CLIMEX parameters and values, number of records used in the analysis, and model accuracy for the four woody (first four) and three herbaceous dicots (last three) biofuel feedstock crops.

Parameter	Description	*Eucalyptus globulus*	*Jatropha curcas*	*Paulownia tomentosa*	*Triadica sebifera*	*Nicotiana tabacum*	*Medicago sativa*	*Pueraria montana*
DV0	Limiting low temperature	8°C	15°C	8°C	12°C	10°C	8°C	10°C
DV1	Lower optimal temperature	14°C	20°C	12°C	24°C	12°C	15°C	16°C
DV2	Upper optimal temperature	32°C	33°C	30°C	35°C	33°C	26°C	30°C
DV3	Limiting high temperature	38°C	36°C	35°C	40°C	36°C	30°C	35°C
SM0	Limiting low soil moisture	0.1	0.35	0.1	0.125	0.2	0.1	0.1
SM1	Lower optimal soil moisture	0.3	0.7	0.3	0.25	0.7	0.2	0.3
SM2	Upper optimal soil moisture	1.2	1.5	1	2	1	1	1
SM3	Limiting high soil moisture	2	2.5	2	3	2	2	2
TTCS	Cold stress temperature threshold	0°C	2°C	0°C	−3°C	−4°C	-	0°C
THCS	Cold stress temperature rate	−0.005	−0.0001	−0.0005	−0.007	−0.0003	-	−0.0005
TTHS	Heat stress temperature threshold	-	37°C	-	42°C	40°C	35°C	-
THHS	Heat stress temperature rate	-	0.0002	-	0.005	0.0002	0.01	-
SMDS	Dry stress threshold	0.01	0.1	0.01	0.2	0.01	0.01	0.01
HDS	Dry stress rate	−0.003	−0.001	−0.007	−0.005	−0.001	−0.001	−0.007
SMWS	Wet stress threshold	-	2.5	-	2	4	-	-
HWS	Wet stress rate	-	0.002	-	0.002	0.002	-	-
TTHW	Hot-wet degree-day threshold	30°C	-	30°C	-	36°C	-	-
MTHW	Hot-wet moisture threshold	1	-	1	-	0.7	-	-
PHW	Hot-wet stress accumulation rate	0.075	-	0.075	-	0.075	-	-
	Total number of records (*N*)	703	394	531	312	318	25,345	957
	EI≥30 (% total)	98.4%	70.3%	96.2%	96.8%	82.1%	90.5%	90.7%
	EI≥20 (% total)	99.3%	97.7%	99.2%	96.8%	94.3%	95.6%	97.1%
	EI≥20 “water subsidy” (% total)	-	98.0%	-	98.1%	-	98.6%	-

**Table 3:** CLIMEX parameters and values, number of records used in the analysis, and model accuracy for the nine invasive species, including one dicot (*Cannabis sativa*) and eight perennial grasses.

Parameter	*Cannabis sativa*	*Cynodon dactylon*	*Dactylis glomerata*	*Elytrigia repens*	*Imperata cylindrica*	*Pennisetum clandestinum*	*Phalaris aquatica*	*Schedonorus phoenix*	*Sorghum halepense*
DV0	5°C	12°C	5°C	5°C	10°C	10°C	8°C	5°C	5°C
DV1	12°C	15°C	10°C	10°C	16°C	14°C	13°C	12°C	20°C
DV2	27°C	33°C	26°C	28°C	30°C	31°C	27°C	27°C	30°C
DV3	30°C	38°C	30°C	30°C	35°C	35°C	30°C	30°C	35°C
SM0	0.1	0.1	0.1	0.1	0.1	0.1	0.1	0.1	0.01
SM1	0.4	0.2	0.2	0.2	0.3	0.4	0.4	0.4	0.1
SM2	1	1	1	1	1	1.5	1	1	1.5
SM3	10	10	10	1.5	2	10	10	4	2
TTCS	0°C	5°C	-	-	0°C	0°C	3°C	−3°C	2°C
THCS	−0.0001	−0.0001	-	-	−0.005	−0.01	−0.001	−0.0002	−0.0001
TTHS	40°C	-	35°C	32°C	-	38°C	-	40°C	40°C
THHS	0.002	-	0.01	0.01	-	0.0002	-	0.002	0.005
SMDS	0.02	-	0.01	0.01	0.01	0.02	0.02	0.02	0.01
HDS	−0.005	-	−0.001	−0.001	−0.007	−0.0003	−0.05	−0.005	−0.005
SMWS	-	-	-	2.5	-	-	-	-	-
HWS	-	-	-	0.002	-	-	-	-	-
TTHW	-	-	30	32	-	30°C	-	-	-
MTHW	-	-	1	1	-	0.8	-	-	-
PHW	-	-	0.07	0.002	-	0.075	-	-	-
*N*	3163	8854	52242	58115	717	255	679	24978	2628
EI≥30	92.8%	84.6%	98.1%	97.7%	62.9%	80.0%	83.8%	98.6%	85.7%
EI≥20	97.0%	92.6%	99.3%	99.1%	87.4%	91.8%	96.2%	99.2%	96.5%
EI≥20+water	98.3%	97.8%	99.5%	-	96.2%	87.5%	-	99.8%	98.4%

**Figure 1:**
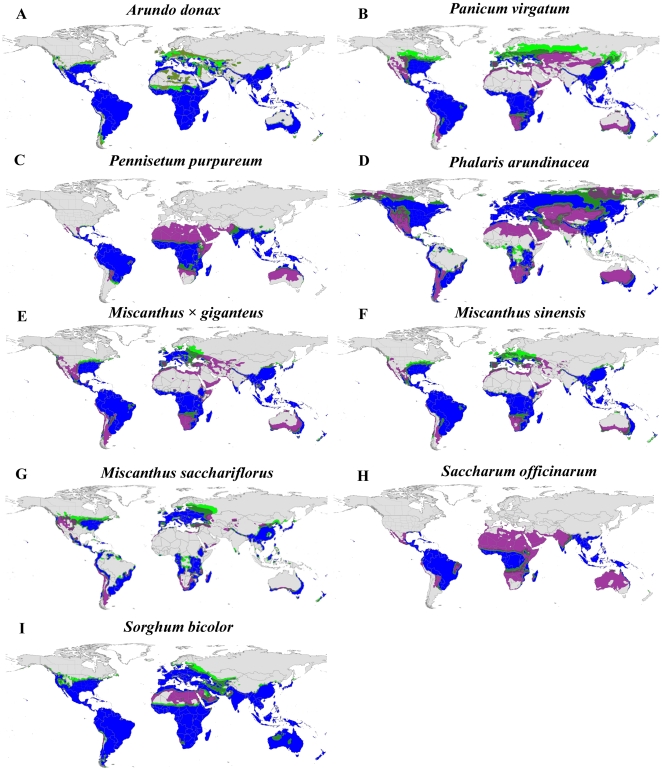
Climate suitability maps for nine grass candidate biofuel feedstocks. Some of the species (A, C, D, F, G) are known weeds of the US, others (B) are native, and some (E, H, I) are currently under cultivation. The colors represent the CLIMEX ecoclimatic index (EI) where gray (EI≤10) is ‘unfavorable’, light green (11>EI>20) is ‘suitable’, dark green (21>EI>30) is ‘favorable’, and blue (EI≥31) is ‘very favorable’. The purple regions are those with an EI>20 when a permanent water source is available.

**Figure 2:**
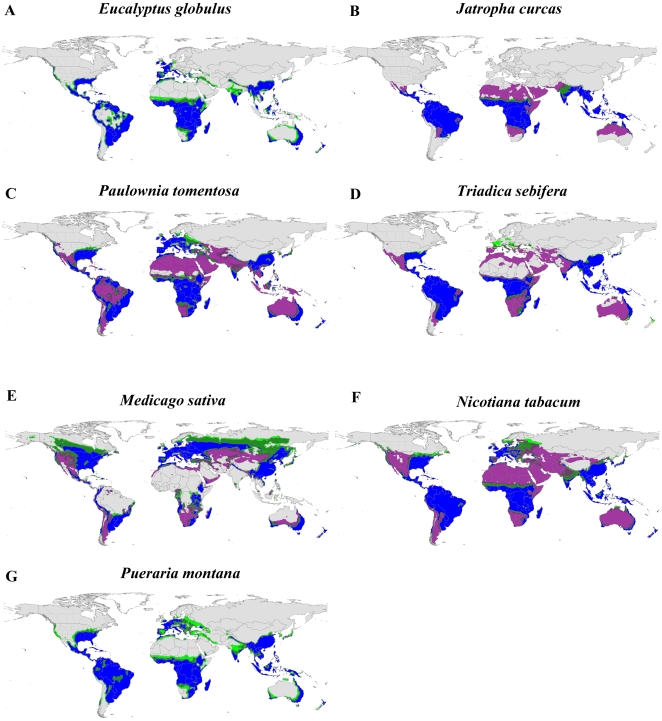
Climate suitability maps for four woody and three herbaceous candidate biofuel feedstocks. Some of the species (A, C, D, G) are known weeds of the US, and some (B, E, F) are currently under cultivation. The colors represent the CLIMEX ecoclimatic index (EI) where gray (EI≤10) is ‘unfavorable’, light green (11>EI>20) is ‘suitable’, dark green (21>EI>30) is ‘favorable’, and blue (EI≥31) is ‘very favorable’. The purple regions are those with an EI>20 when a permanent water source is available.

**Figure 3:**
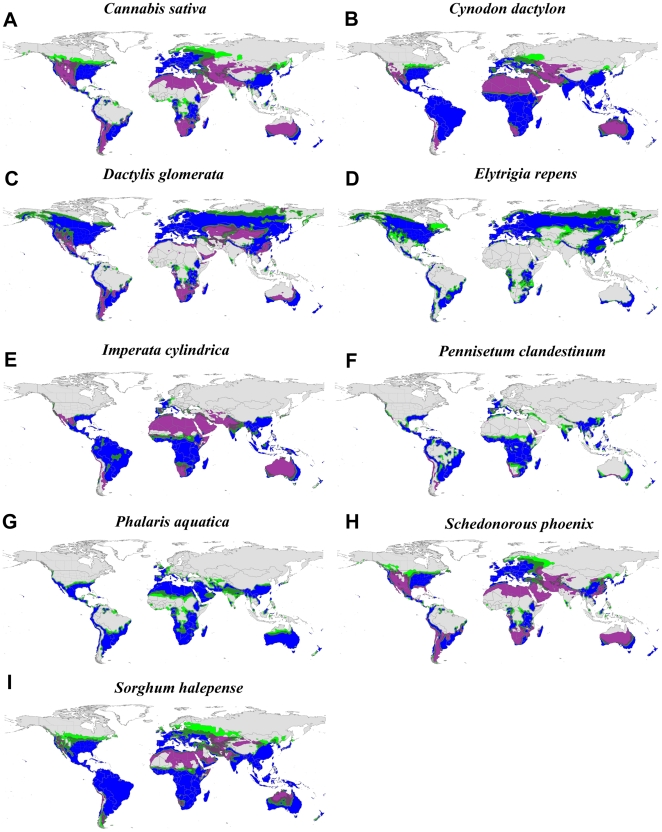
Climate suitability maps for nine invasive species of agronomic origin. All taxa (A–I) are currently weedy species in the US. The colors represent the CLIMEX ecoclimatic index (EI) where gray (EI≤10) is ‘unfavorable’, light green (11>EI>20) is ‘suitable’, dark green (21>EI>30) is ‘favorable’, and blue (EI≥31) is ‘very favorable’. The purple regions are those with an EI>20 when a permanent water source is available.

The suite of potential bioenergy feedstocks we investigated demonstrates a vast range of potentially cultivatable land across the globe both with and without irrigation inputs (Figs. 1, 2). With few exceptions (e.g., *S. officinarum*, *P. purpureum*), the bioenergy crops exhibit broad climatic tolerance, which allows tremendous flexibility in choosing crops, especially in areas with high summer rainfall and long growing seasons (e.g., southeastern US, Amazon Basin, eastern Australia). Unsurprisingly, the invasive species of agronomic origin have very similar global climate niche profiles as the proposed bioenergy crops (Fig. 3), also demonstrating broad climatic tolerance. The “perennial water scenario”, which mimics both irrigation additions as well as access to a permanent water supply [18], typically expanded the climate niche to regions that are arid during the growing season, but are otherwise suitable: western US, northern Africa, central and western Australia, and the Middle East (Figs. 1, 2, 3).

**Figure 4:**
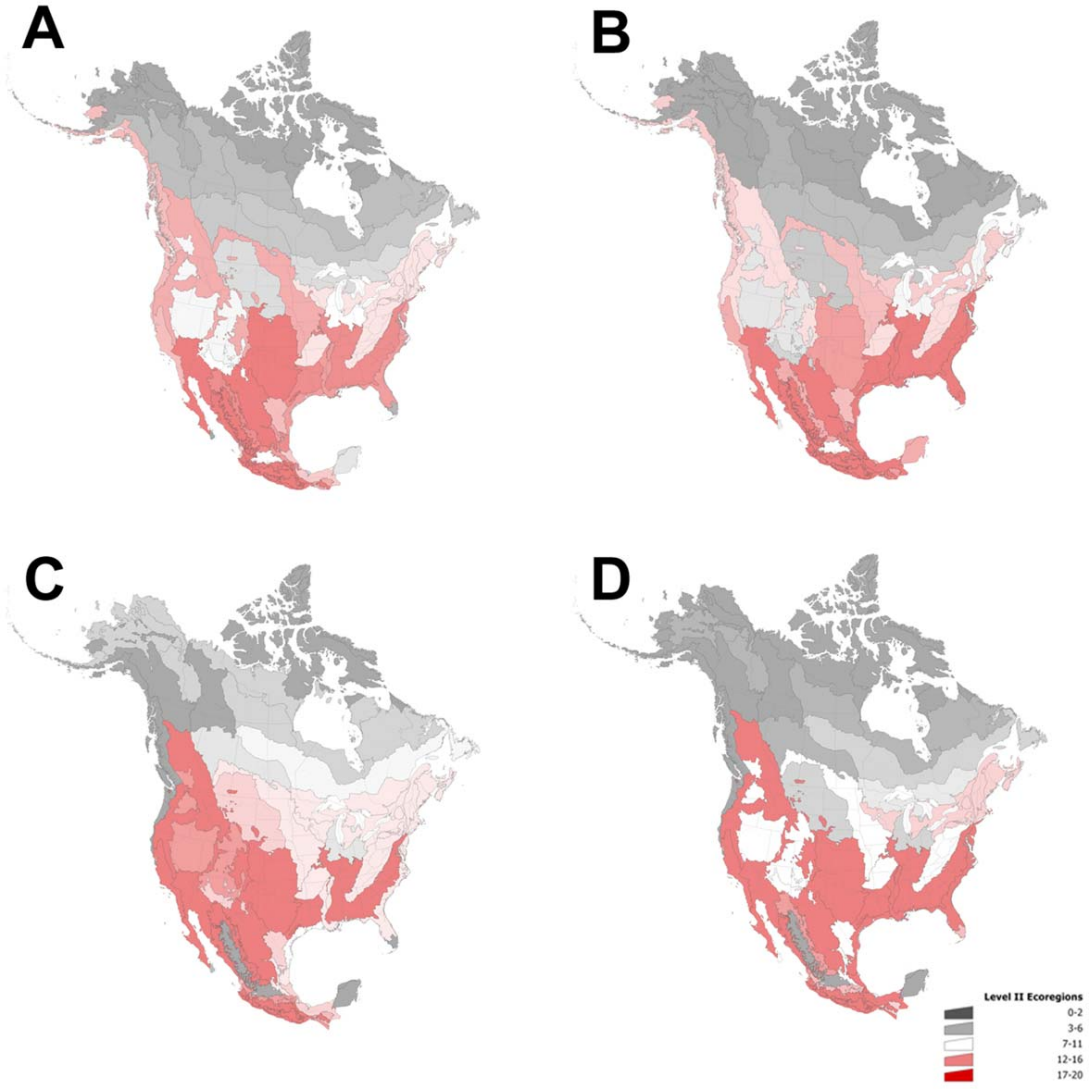
Ecoregion climatic suitability of biofuel crops and invasive species. Potential biofuel crops with (A) moderate and (B) high suitability in contrast to existing invasive species with (C) moderate and (D) high suitability.

**Figure 5:**
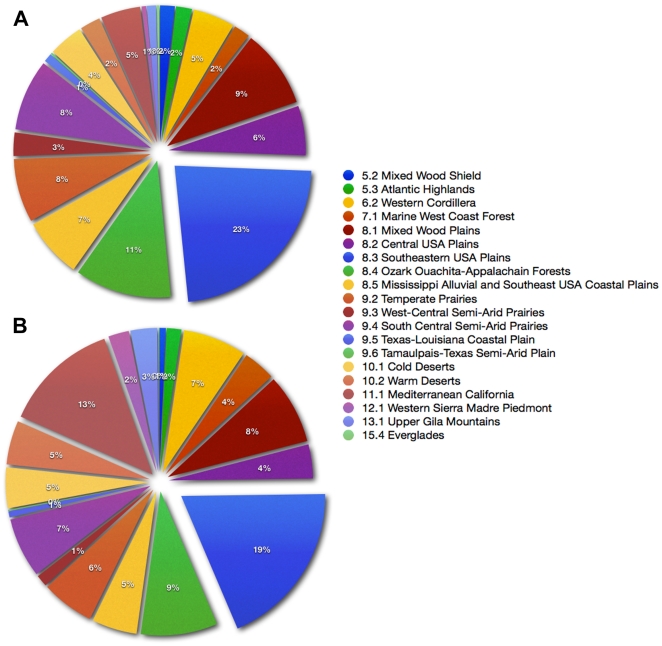
Distribution of populations in ecoregions of the continental US. Proportion of (A) 15 proposed biofuel crops and (B) nine invasive species located in each ecoregion. The ecoregions begin with 5.2 Mixed Woods Shield at the 12 o'clock position and proceed clockwise according to the legend.

The ecoregional evaluation of bioenergy crops and known invasive species demonstrates tremendous overlap at both high (EI≥30) and moderate (EI≥20) climate suitability (Figs. 4, 5). The southern and western US ecoregions support the greatest number of invasive species of agronomic origin, especially the Southeastern USA Plains, Mixed Woods Plains, and Mediterranean California (Fig. 5B). This differs only slightly for bioenergy crops with the Southeastern USA Plains, Mixed Woods Plains, and Western Sierra Madre Piedmont ecoregions supporting the most taxa (Fig. 5A). Bioenergy crops had a high climate match (EI≥30) in at least some part of 20–85% of US ecoregions without a permanent water supply, and 35–90% with an irrigation factor (Table 4). The invasive species had a high climate match in 50–85% without a permanent water source, and 60–90% with permanent water source.

**Table 4:** Ecoregional distribution of biofuel crops and invasive species.

Species	% North American Ecoregions		% Continental US Ecoregions	
	Standard	+Water	Standard	+Water
**Biofuels**				
*Arundo donax*	-	62%	-	85%
*Eucalyptus globulus*	50%	-	55%	-
*Jatropha curcas*	38%	48%	20%	35%
*Medicago sativa*	64%	66%	85%	90%
*Miscanthus sacchariflorus*	56%	-	75%	-
*M. sinensis*	56%	64%	60%	85%
*Nicotiana tabacum*	60%	-	70%	-
*Panicum virgatum*	56%	66%	55%	90%
*Paulownia tomentosa*	58%	-	75%	-
*Pennisetum purpureum*	40%	46%	25%	35%
*Phalaris arundinacea*	80%	70%	80%	55%
*Pueraria montana*	56%	-	65%	-
*Saccharum officinarum*	32%	50%	20%	45%
*Sorghum bicolor*	28%	36%	60%	75%
*Triadica sebifera*	42%	44%	40%	60%
**Invasives**				
*Cannabis sativa*	58%	54%	75%	70%
*Cynodon dactylon*	56%	52%	80%	80%
*Dactylis glomerata*	74%	72%	75%	80%
*Elytrigia repens*	78%	-	85%	-
*Imperata cylindrica*	50%	52%	50%	65%
*Pennisetum clandestinum*	46%	38%	50%	35%
*Phalaris aquatica*	-	48%	-	60%
*Schedonorus phoenix*	54%	64%	70%	95%
*Sorghum halepense*	62%	-	80%	-

Percentage of ecoregions in North America (n = 50) or the Continental US (n = 20) that have some portion of the current or predicted (CLIMEX EI≥30) range of each species within its boundaries.

## Discussion

The climate niche for the bioenergy crops evaluated demonstrates that temperate to sub-tropical regions of the world that receive consistent summer rainfall and have a warm/hot summer and a long growing season will be most favorable, and will provide the greatest number of feedstock choices without the need for consistent summer irrigation. The most favorable regions include the southeastern and southcentral US, the Amazon basin, sub-Saharan and central Africa, western continental Europe, southeast Asia, and eastern Australia. In North America, the ecoregions that appear most suitable for bioenergy crops are the Southeastern USA Plains characterized by weakly developed soils, average annual temperatures of 13–19 C, and 1000–1600 mm of annual precipitation (23% of the databased populations), the Ozark Ouachita-Appalachian Forests characterized by weakly developed soils, average annual temperatures of 17–18 C, and 1000–2000 mm of annual precipitation (11% of databased populations), and the Mixed Woods Plains characterized by forest and fine textured soils, average annual temperatures 4–10 C, and 720–1200 mm of annual precipitation (9% of databased populations). The large number of collected populations in these ecoregions suggests that many of these bioenergy crops are already established, indicating high climatic suitability, as well as favorable abiotic and biotic conditions (locally at least). The USDA recently released an analysis demonstrating that the US Southeast will likely yield about 50% of the biomass needed to meet the Renewable Fuel Standard. Our analysis demonstrates that this region will have the greatest number of species from which to choose.

The global climate niche distributions for the invasive species of agronomic origin were generally very similar to the bioenergy crops, except for the sub-tropical *Pennisetum clandestinum*. Many of these weedy species continue to be utilized as turfgrass (*S. phoenix*, *Cynodon dactylon*) or forages (*D. glomerata*, *E. repens*, *S. phoenix*), and may be under irrigation, which greatly expands their climate niche because cultivation generally reduces environmental stochasticity [20]. Coincidently, the ecoregions that have the greatest number of invasive species populations are nearly identical to those for bioenergy crops, except for the Mediterranean region of California, which is one of the most heavily invaded regions of the US [29]. However, this arid environment is unlikely to be a major location for bioenergy crop production, due to the requirement for summer irrigation—currently a scarce resource in the western US [30].

Broad climatic tolerance, or a large climate niche, is positively correlated with invasiveness, as this greatly increases the probability of surviving outside of cultivation in the multitude of possible environments that might be encountered. However, this is also a desirable character of crop plants by increasing the suitable agro-ecoregions for cultivation. Therefore, it is not surprising that the climate niche for plants with an agronomic origin are large, as breeders generally select for this characteristic, and often direct efforts to enhance cold, heat, or drought tolerance, which broadens the climate niche [31]. Additionally, the boundaries of the climate niche can be used to impose functional sterility on bioenergy crops, which increases yield while simultaneously reducing the escape potential by precluding seed production [32]. The fact that the bioenergy crops investigated here have similarly broad climatic tolerance as the invasive species in no way indicates eventual invasiveness. Nevertheless, this characteristic—broad climatic tolerance—should be considered when evaluating the risk of invasiveness for each bioenergy species [10].

The climate niche of bioenergy crops must be accounted for when evaluating the invasion risk, and should not be assumed to be a high match as has previously occurred [10], [13], [14]. Additionally, evaluating the climate niche for introduced species should not occur at the continental or national geopolitical scale as is current practice in existing risk assessment frameworks [16]. Large-scale assessments that cover vast geographic regions with diverse climates are prone to overestimating the risk of invasion because the probability of at least one propagule encountering one susceptible community is extremely high. Pheloung and colleagues recognized the importance of evaluating the climate match for target species [16], but performed their assessments at the continental scale for Australia, which has regions varying from deserts to tropical rain forests, so the likelihood of at least one habitat having a high climate match for the country is nearly certain. An additional consideration is that populations of species are invasive, not the species themselves. For example, *Arundo donax* is a state-listed noxious weed in California and Texas where it dominates riparian habitat [29]. However, *Arundo* is only occasionally found in the Mid-Atlantic and Southeastern US where it has existed for many decades [10]. Therefore, *Arundo* may be benignly cultivated in some areas of the US, while being a noxious weed in other areas of the US. This relates to the spatial context of a risk assessment in that invasiveness occurs on a spatial scale smaller than countries, and should not be restricted to ecologically arbitrary geopolitical boundaries.

In an attempt to address the need for evaluating invasive risk at sub-national levels we incorporated an ecoregional assessment of the climate niche. There are several ecoregional designations for North America available that vary in spatial context: Level I contains 15 broad categories, Level II has 50 smaller categories, while Level III contains 182 categories. We chose the Level II designation as it provides 20 distinct ecoregions in the US that the species of interest occur, which captures sufficient variation in climate, ecosystems, and land use to be useful for stakeholders without being too general (Level I) or too specific (Level III). Some collected populations of both bioenergy crops and invasive species occurred in all 20 ecoregions, though the relative distribution of these populations was extremely unbalanced (Fig. 5), with the Southeastern US supporting the greatest number of populations.

As the bioeconomy grows globally, especially in the southeastern US, which is estimated to support about 50% of the biomass to meet federal mandates [4], precaution should be taken in large-scale introductions of potentially invasive bioenergy crops. This mistake has been made in the past by federally subsidized large-scale adoption of novel species that ultimately turn out costing orders of magnitude more taxpayer dollars to manage (eg, kudzu and johnsongrass).
